# Residual Stress Control Using Process Optimization in Directed Energy Deposition

**DOI:** 10.3390/ma16196610

**Published:** 2023-10-09

**Authors:** Manping Cheng, Xi Zou, Yang Pan, Yan Zhou, Wenyang Liu, Lijun Song

**Affiliations:** 1 Research Institute of Automobile Parts Technology, Hunan Institute of Technology, Hengyang 421002, China; chengmanping@hnit.edu.cn; 2 State Key Laboratory of Advanced Design and Manufacturing for Vehicle Body, Hunan Provincial Key Laboratory of Intelligent Laser Manufacturing, Hunan University, Changsha 410082, China; liuwenyang@hnu.edu.cn; 3 School of Mechanical Engineering, Hunan University of Science and Technology, Xiangtan 411201, China; 4 School of Information Engineering, Hubei University of Economics, Wuhan 430205, China; yanzhou626@gmail.com

**Keywords:** additive manufacturing, thermodynamic response, 316L stainless steel, process parameters, residual stress

## Abstract

This paper mainly analyzes the typical thermodynamic response (thermal history, thermal strain and residual stress) in a conventional continuous-wave (CW) laser during Directed Energy Deposition (DED). The influence of process parameters (laser power and scanning speed) on the temperature gradient in the heat-affected zone, thermal strain and residual stress are studied, and the corresponding relationship are established. The results show that a reduction in residual stress can be obtained by decreasing the temperature gradient. However, the method of reducing the temperature gradient by changing process parameters leads to low forming quality and low density. A pulse-wave laser (PW) is proposed to actively control the residual stress of the deposited sample. This laser mode can reduce not only the temperature gradient in the process of DED but also the in situ release of thermal stress, correspondingly greatly reducing the residual stress.

## 1. Introduction

The forming process of DED is a thermal mechanical coupling process [1,2], and its thermodynamic response strongly depends on the thermal behavior of the molten pool [3]. The non-uniformity of heat distribution in the molten pool during the machining process leads to a great temperature gradient [4,5]. Due to the constraint of the surrounding cold metal substrate, a great thermal strain is formed in the solid-state region. It is left in the metal deposited sample in the form of residual stress after cooling [6,7,8]. In many cases, the existence of residual stress reduces the strength of the component, resulting in brittle fracture and stress corrosion. Therefore, improving the process parameters in DED is one of the key factors to reduce the residual stress of the deposited sample. The effects of laser power and scanning speed on residual stress were studied [9,10]. However, residual stress could only be reduced by up to 20% with the method of changing scanning speed and power [11]. On this basis, Amanda [12] et al. concluded that the continuous scanning strategy can significantly reduce the residual stress by 39% compared to the island scanning strategy for 316L stainless steel. On the contrary, Michael [13] et al., concluded that the island scanning strategy in tool steels could reduce the residual stresses by 30%. Erik R. Denlinger [14] investigated the effect of inter-layer dwell time on the deformation and residual stresses in Ti-6Al-4V and Inconel 625, and stated that for Inconel 625 material, increasing the dwell time was beneficial in reducing the deformation and residual stress (about 23%). On the contrary, for Ti-6Al-4V material, the decrease in dwell time leads to a significant decrease in residual stresses and cumulative deformation (about 55%). Meanwhile, preheating is another common method to reduce the residual stress. Pruk [15] used a localized preheating process to reduce the residual stresses by 30% in rectangular thin-walled 304 stainless steel parts. Similarly, Shiomil [16] et al. concluded that heat treatment, laser re-scanning and substrate preheating are important methods to reduce residual stresses in Cr-Mo steels (70, 55 and 40%, respectively). The reduction in residual stresses in the deposited sample by optimization of process parameters and preheating are all achieved by reducing the temperature gradient during the DED process.

However, these methods have some limitations, the reduction in the temperature gradient caused by optimization of process parameters has a limited effect on the reduction in residual stresses, and the fabrication time increases accordingly with the reduction in the energy thermal input, which is detrimental to the improvement of the processing efficiency. Inappropriate laser energy density leads to porosity defects, resulting in the insufficient densification of the deposited sample [17]. Multiple processes are required by pre-treatment methods, such as preheating, which increases manufacturing costs, wastes manufacturing time, and reduces manufacturing efficiency. Furthermore, although many methods to control and reduce the residual stress of the deposited sample have been given by the above research, the mechanisms have still been rarely studied.

In the work reported here, the effects of the DED process parameters (laser power and scanning speed) on the thermodynamic response (temperature gradient and thermal strain) of continuous-wave (CW) laser and the residual stress of the deposited sample was studied. The correlation between temperature gradient, thermal strain, and residual stress were established. The problem of excessive residual stress in the deposited sample can be solved by reducing the temperature gradient and thermal strain though changing laser power and scanning speed. However, it brings new problems of low forming quality and low densification. On this basis, a pulsed-wave (PW) laser, with a periodic heating and cooling cycle, was proposed, which would reduce the residual stress of deposited sample effectively while ensuring the same densification and forming quality as a CW laser.

## 2. Materials and Methods

**Figure 1:**
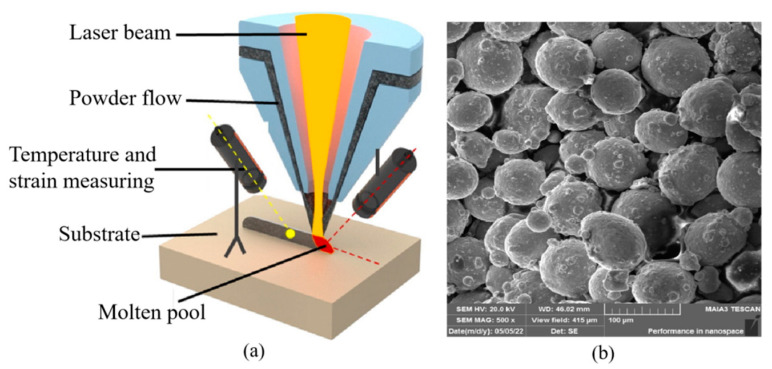
(**a**) Schematic diagram of DED processing. (**b**) SEM image of 316L stainless steel powder.

**Figure 2:**
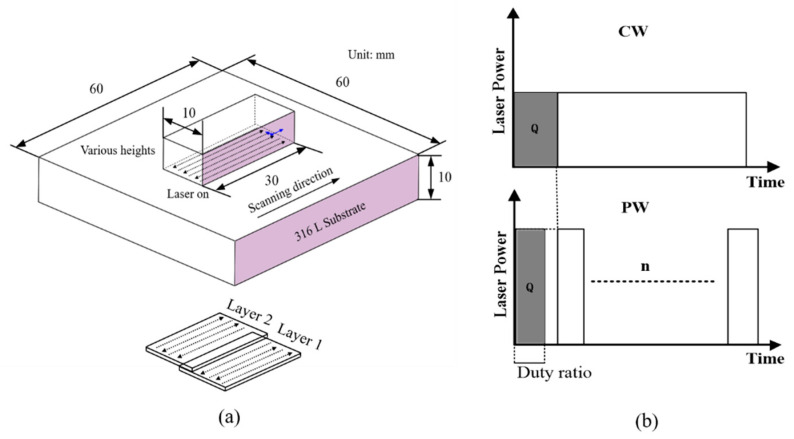
(**a**) Sample size and scanning path. (**b**) Schematic diagram of continuous-wave (CW) laser and pulsed-wave (PW) laser.

Samples were fabricated using a YLS-5000-CL laser direct energy deposition system with a 1070 nm wavelength fiber laser. The Schematic diagram of DED processing is shown in Figure 1a. Metal or alloy powders were irradiated by high energy laser beams, and then melted into a liquid state by utilizing the high energy density and high focus of the laser beam. Then, it rapidly solidify into a solid state and is deposited at a specific location, thus achieving three-dimensional object construction. Commercially available 316L stainless steel powders were used. The SEM morphology image was shown in Figure 1b. The median particle size of the powder was 89.44 μm. They were baked in a vacuum drying chamber at 120 °C for 20 min in order to remove the moisture. The processing parameters are listed in Table 1. Other process parameters are as follows: the beam diameter is 1.2 mm, the powder feeding rate is 11.5 g/min, the overlap rate is 50%, and the shielding gas is pure argon used at a rate of 10 L/min. Each sample deposits 30 layers with a thickness of 0.2 mm, ultimately forming a block with a width of 10 mm and a length of 40 mm. The deposition height varies with the process parameters. The distance between the deposition head and the substrate is 14 mm to ensure that the laser and powder converge at one point on the substrate. The scanning strategy is horizontal back-and-forth linear scanning, as shown in Figure 2a. The peak laser power was set to 800 W for PW mode, with the corresponding scanning speed at 8 mm/s, duty cycle of 75%, and frequency of 10 Hz, as shown in Figure 2b. It aims to ensure the consistency of the energy input of S9 (PW) and S3 (CW). The temperature distribution during DED was measured using an FLIR A615 infrared thermal imager, then the temperature gradient was calculated using the method mentioned in Refs [18], the spatial temperature data along the laser scanning direction were extract, and the temperature gradient data were calculated using MATLAB, with a resolution of 640 × 480, sampling frequency of 50 Hz, the measurement accuracy of ±2 °C. DED 316 L samples for residual stress were measured by the contour method, using envelope processing, averaging the left and right contours, Gauss mixed model simulation, and node interpolation processing. The final deformation contour value was obtained, and it was loaded back into the stress reconstruction finite element model as a boundary condition to obtain the stress field of the tested surface. A detailed description is given in Refs [19,20], and the measurement accuracy was 28 MPa [21]. An industrial camera and the blue-assisted light source with digital image correlation method (DIC) [22,23] was used to monitor in situ thermal strain with 0.1% precision. The image resolution was 640 × 480, and the image acquisition frequency was set to 0.05 Hz. Deposited samples were separated from the substrate by wire cutting, and then the Archimedes drainage method was used to measure the density of deposited samples. The thermal stress evolution of deposited samples during DED was simulated by the commercial finite element software ANSYS 15.

## 3. Results

### 3.1. Temperature Gradient in Solid Region

**Figure 3:**
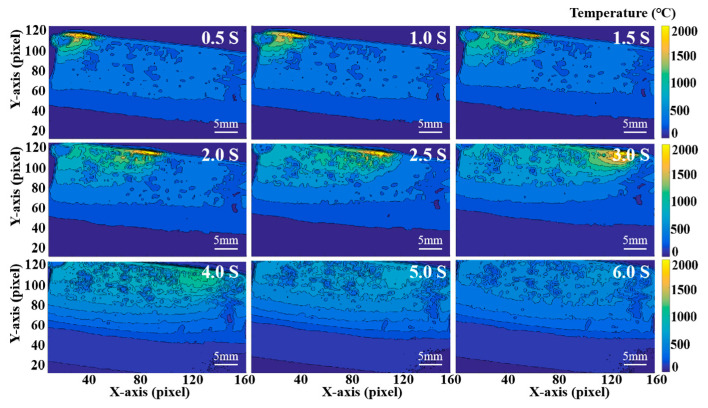
Time-dependent temperature nephogram of S3 sample during deposition.

A time-dependent temperature map of S3 sample during deposition is shown in Figure 3. The upper right corner is the deposition time, in which 0.5~3.0 s is the heating stage and 4.0~6.0 s is the cooling stage. At the optional time (0.5~1.0 s) in the initial heating stage, the laser acts on the front end of the deposited sample to quickly form a molten pool, and the temperature in the center of the molten pool reaches 2200 °C. The temperature rapidly diffuses from the center of the molten pool to both sides, forming a temperature gradient due to heat conduction. The molten pool moves forward with the movement of the laser heat source. Due to the slow cooling rate of the metal material in front of the deposited sample, the molten pool gradually forms a “tailing” phenomenon at the middle (1.5~2.0 s) and later stage (2.5~3.0 s) of heating time.

**Figure 4:**
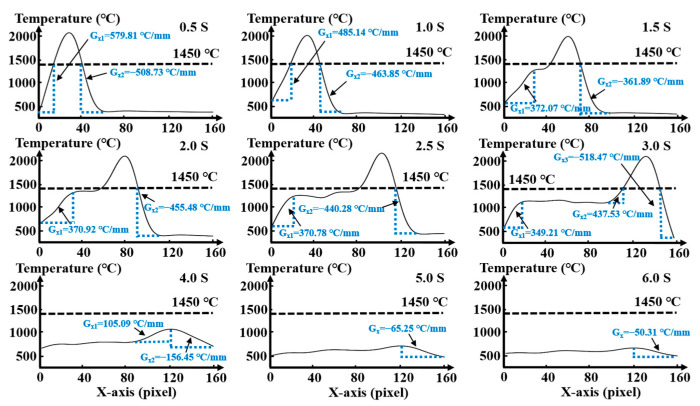
Time-dependent fitting temperature curve and temperature gradient of S3 sample during deposition.

A time-dependent fitting temperature curve in the laser scanning direction (longitudinal) and temperature gradient in solid region of S3 sample during deposition is shown in Figure 4. The black dotted line represents the melting point (1450 °C) of 316L stainless steel, which is used to distinguish the liquid and solid state of metals. The blue dotted line area is the temperature gradient in solid region of the deposited sample. The temperature gradient at the rear end of the molten pool is positive, and at the front end is negative. Due to the advance of the molten pool, the temperature gradient at the front of the molten pool in the last second would be clad by the molten pool in the next second, which has no effect on the formation of thermal stress. Therefore, only the temperature gradient at the rear of the molten pool is considered in this paper. At the initial stage of heating (0.5~1.0 s), the temperature presents a “parabolic” distribution. It leads to a high temperature gradient in the solid region at the rear of the molten pool, reaching 579.81 °C/mm at 0.5 s, 485.14 °C/mm at 1.0 s. The average temperature gradient at two times is 532.47 °C/mm. Over time, the molten pool gradually moves, and a small area with stable temperature gradient appears at the end of the molten pool in the middle heating period (1.5~2.0 s). In this area, the continuous heat conduction and heat dissipation of the molten pool keep in balance, which makes the temperature gradient in this area is low. However, at the front of the deposited sample, its position is far from the molten pool, and the continuous heat conduction of the molten pool cannot make up for the heat loss. This results in a large temperature gradient, with an average value of 370.85 °C/mm. At the later stage of heating (2.5~3.0 s), the temperature stability at the end of the molten pool is more obvious. The average temperature gradient at the front of the deposited sample remains at 359.99 °C/mm. In the cooling stage (4.0~6.0 s), the peak temperature decreases with time until room temperature, and the temperature gradient gradually decreases from 105.09 °C/mm to 50.31 °C/mm. It can be seen that during the movement of the laser, the temperature gradient in the solid region of the deposited sample gradually decreases until it is stable.

**Figure 5:**
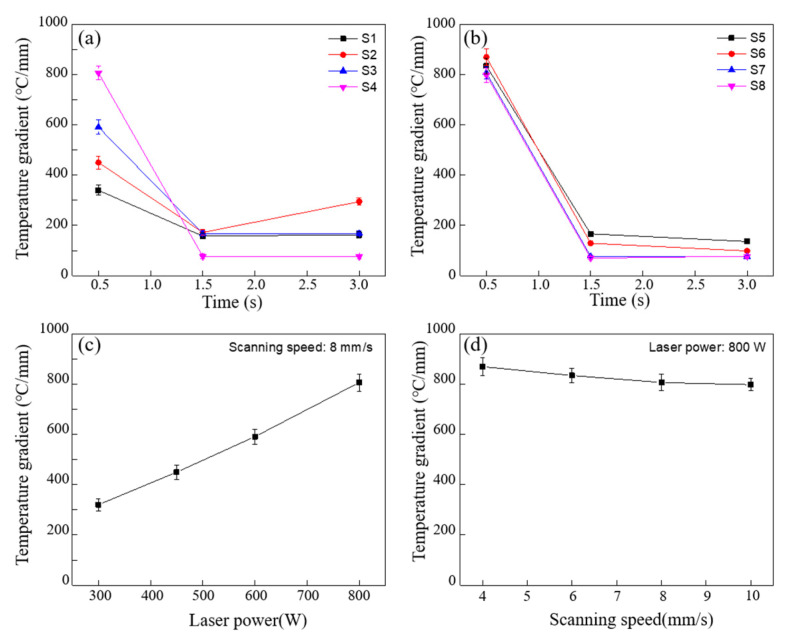
Time-dependent temperature gradient under different laser process parameters. (**a**,**b**) the law of time-dependent temperature gradient in solid region under different laser power and scanning speed; (**c**,**d**) maximum temperature gradient in solid region under different laser power and scanning speed at 0.5 s.

The law of time-dependent temperature gradient in front of the deposited sample under different laser power and scanning speed during deposition is shown in Figure 5a,b. At the initial time of deposition (0.5 s), the metal in front of the deposited sample is heated rapidly, resulting in a large temperature difference with the surrounding cold metal, a large temperature gradient is formed. As the laser moves to the middle of the deposition layer (1.5 s), the metal at the front of the deposited sample has no time to cool and maintain a high temperature, while the temperature in the middle of the deposited sample rises rapidly due to the arrival of the heat source. At this time, the temperature difference from the front to the middle of the deposited sample shrinks expeditiously, which lead to a sharp decline in the temperature gradient in solid region. Similarly, at the last moment of deposition (3 s), the laser moves to the end of the deposited sample, the front metal material has sufficient time to cool, the temperature falls, and the temperature gradient in solid region remains at a low level.

Special attention should be paid to the change of temperature gradient in solid region at different laser power (scanning speed of 8 mm/s) and scanning speed (laser power of 800 W) at 0.5 s, as shown in Figure 5c,d. As the laser power increases from 300 W to 800 W, the temperature gradient in the solid region increases linearly from 320.2 °C/mm to 806.4 °C/mm, and correspondingly decreases slowly from 869.4 °C/mm to 797.8 °C/mm when the scanning speed increases from 4 mm/s to 10 mm/s. These results show that lower laser power and faster scanning speed contribute to obtaining a lower temperature gradient in the solid region. Meanwhile, compared with the scanning speed, laser power is the main factor affecting the temperature gradient.

### 3.2. Thermal Strain

**Figure 6:**
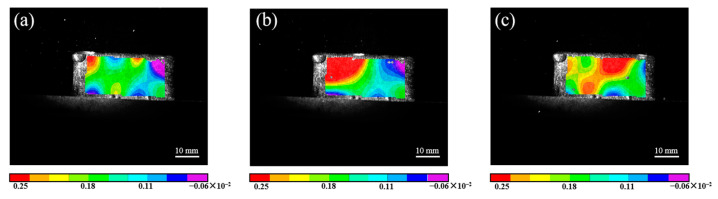
Thermal strain nephogram of S3 sample during deposition: (**a**) 0.5 s; (**b**) 1.5 s; (**c**) 3.0 s.

The time-dependent map of thermal strain during deposition is shown in Figure 6. At the initial time (0.5 s), the maximum thermal strain appears at the front of the deposited sample. The strain in this area shows a large positive strain (red area), i.e., it reaches about 0.25%, while the thermal strain in other areas is negative so as to balance the strain of the whole surface (purple and blue areas). With the movement of the laser heat source, the heat accumulation increases, resulting in a larger positive strain region at the intermediate time (1.5 s). it always exists at the rear of the molten pool, which is caused by the strong temperature gradient and the constraints of the surrounding cold metal [24]. When the laser moves to the end (3.0 s), the thermal strain at the front of the deposited sample decreases slightly to 0.18%. Because the heat source is far away from the region, the temperature gradient is reduced, and correspondingly, the metal material is cold contracted, resulting in the recovery of the elastic thermal strain. The remaining unrecoverable strain is stored in the deposited sample in the form of plastic deformation. Meanwhile, the maximum thermal strain region appears at the end of the deposited sample.

**Figure 7:**
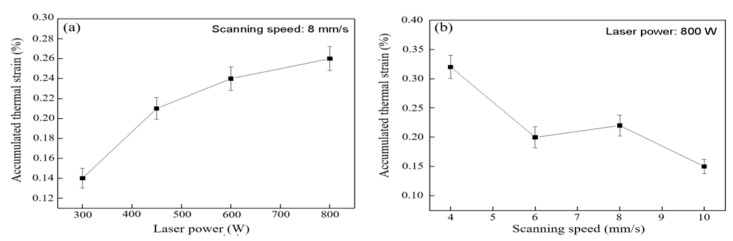
Influence of process parameters on accumulated thermal strain: (**a**) laser power; (**b**) scanning speed.

The maximum longitudinal thermal strain under different laser power (scanning speed 8 mm/s) and scanning speed (laser power 800 W) is shown in Figure 7. With the laser power increasing from 300 W to 800 W, the maximum longitudinal thermal strain increases from 0.14% to 0.26%. On the contrary, the increase in scanning speed (4 mm/s~10 mm/s) is conducive to the reduction in thermal strain (0.35%~0.12%). The change of laser processing parameters directly affects the temperature gradient and cooling rate of the heat-affected zone. According to the analysis of Figure 5c,d, the temperature gradient in the solid region gradually increases with the increase in laser power and the decrease in scanning speed. It is pointed out that higher laser power and lower scanning speed are conducive to obtaining a slower cooling rate [25]. Under the environment of a large temperature gradient and slow cooling rate, it is more difficult for deposited samples to transfer heat energy through heat conduction and heat radiation. It obtains greater heat accumulation, making the metal continue to expand, which leads to greater thermal strain of the deposited sample. It is worth noting that the difference in the maximum longitudinal thermal strain is small and within the error range at the scanning speeds of 6 mm/s and 8 mm/s. This may be because there is little difference between the temperature gradient and heat-accumulated energy in solid region at these two scanning speeds, and its change trend is consistent with the temperature gradient described in Section 3.1.

### 3.3. Residual Stress

#### 3.3.1. Residual Stress of Thin-Walled Deposited Sample

**Figure 8:**
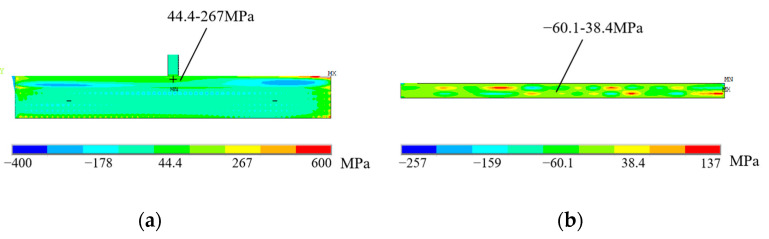
Cloud diagram of residual stress distribution of S3 sample: (**a**) longitudinal residual stress; (**b**) transverse residual stress.

The residual stress of the deposited sample mainly depends on the elastic strain produced by the deposited material in the cooling stage. The residual stress distribution of thin-walled deposited sample (S3 sample) is shown in Figure 8. It is worth noting that a small amount of excessive stress (red area) appears at the edges of the transverse and longitudinal sections of the deposited sample, which is due to the systematic error caused by the inevitable slippage of the CMM probe when measuring the metal edge [26]. Along the horizontal direction, the longitudinal residual stress on the top of the substrate is approximately a parabolic distribution: the middle part is tensile stress (marked as +), and its value range is 44.4~267 MPa; and the two ends are compressive stress (marked as -), and their value range is −400~−178 MPa. Along the depth direction, the longitudinal residual stress reaches the maximum (267 MPa) at the junction of the substrate and the deposited sample. It changes dramatically along the thickness direction of the substrate, forming a large area of compressive stress in the middle of the substrate, with a value of about −66.7 MPa, and a tensile stress of about 44.4 MPa at the bottom. This is caused by the local thermal expansion and contraction of the heat-affected zone and the correspondingly self-balance of the internal stress on the free surface during laser deposition. The transverse residual stress is irregularly distributed on the cross section of the deposited sample, and its value is widely distributed between −60.1~38.4 MPa. The results show that the transverse residual stress of the deposited sample is much smaller than the longitudinal residual stress, which also conforms to the description of literature [27]. Therefore, this paper mainly studies the longitudinal residual stress.

**Figure 9:**
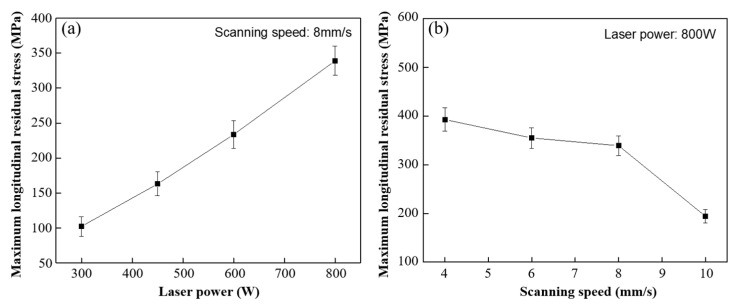
Influence of process parameters on maximum longitudinal residual stress of substrate: (**a**) laser power; (**b**) scanning speed.

The influence law of different processing parameters (laser power and scanning speed) on the maximum longitudinal residual stress of thin-walled deposited sample is shown in Figure 9. Under the condition of fixed scanning speed of 8 mm/s, the maximum longitudinal residual stress of the deposited sample shows a linear increase trend with the increase in laser power, with the minimum of 102.2 MPa under 300 W laser power and the maximum of 339.1 MPa under 800 W. This is due to thermal accumulation, which increases the temperature gradient and thermal strain as the laser power raises, as described in Section 3.1 and Section 3.2. Meanwhile, with a fixed laser power of 800 W, the maximum longitudinal residual stress of the deposited sample gradually decreases with the increase in scanning speed. This is because the faster cooling rate at a high scanning speed can quickly transfer heat through heat conduction, reducing the accumulated heat energy and temperature gradient, resulting in the reduction in thermal strain; this contributes to the reduction in the final residual stress. It is worth noting that the difference in the maximum longitudinal residual stress at the scanning speeds of 6 mm/s and 8 mm/s is small and within the error range. It may be due to the small difference in the temperature gradient and heat accumulated energy at these two scanning speeds, and its change trend is consistent with the temperature gradient and heat strain described in Section 3.1 and Section 3.2.

#### 3.3.2. Residual Stress of Block Deposited Sample

It can be seen from the previous analysis that, relative to the scanning speed, the laser power is the key factor affecting the temperature gradient, thermal strain and residual stress of the deposited sample. Meanwhile, the deposition morphology and forming quality have a great impact on the measurement accuracy of the residual stress of the block deposited. The sample with low density leads to more pores in the material, and the residual stress would be released. Therefore, the prerequisite for measuring the residual stress of the deposited sample is to deposit bulk samples with complete morphology, and high forming quality and density. In the process of DED, different laser peak power has a great impact on the forming quality and density, as shown in Table 2. The forming quality of the S1 sample is poor, and it is obvious that the powder sticking phenomenon occurs at the edge of the deposited sample. Furthermore, its density is only 92.24%, which is caused by the incomplete melting of the powder due to low laser power. For S2–S4 and S9, the deposition morphology and forming quality of the deposited samples are fantastic; the surface is flat without too much sticky powder, and the densities are all about 99.5%. These block-deposited samples meet the requirements of residual stress measurement. However, from Table 2 (S2–S4), it can also be seen that manufacturing efficiency is strongly dependent on laser power. Within the same time, the deposited sample mass and volume decrease with the decrease in laser power. It is obvious that high laser power brings high energy input, resulting in high manufacturing efficiency.

**Figure 10:**
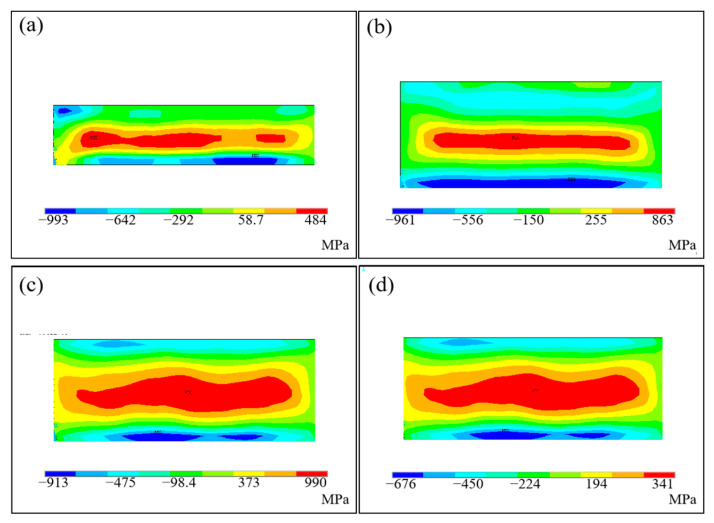
Influence of laser power on longitudinal residual stress of the deposited sample: (**a**) S2 sample; (**b**) S3 sample; (**c**) S4 sample; (**d**) S9 sample.

The longitudinal residual stress distribution of S2–S4 and S9 is shown in Figure 10. There exists high residual tensile stress in the middle of the deposited sample, and the stress is even higher than the yield strength of the material, which can be explained by the triaxial stress state of the residual stress and the strain hardening effect of the material [28,29]. In addition, high temperature reduces the yield strength of the material, turning it from brittle to ductile, and the melting process is considered to be unstable, i.e., the alloy elements with low boiling points are evaporated. This evaporation phenomenon affects the composition of the material, thus changing the mechanical properties of the deposited sample [30]. At the top and bottom of the deposited sample, the corresponding residual compressive stress appears to balance the internal stress on the whole surface. The longitudinal residual tensile stress of bulk deposited samples increases with the increase in laser power.

**Figure 11:**
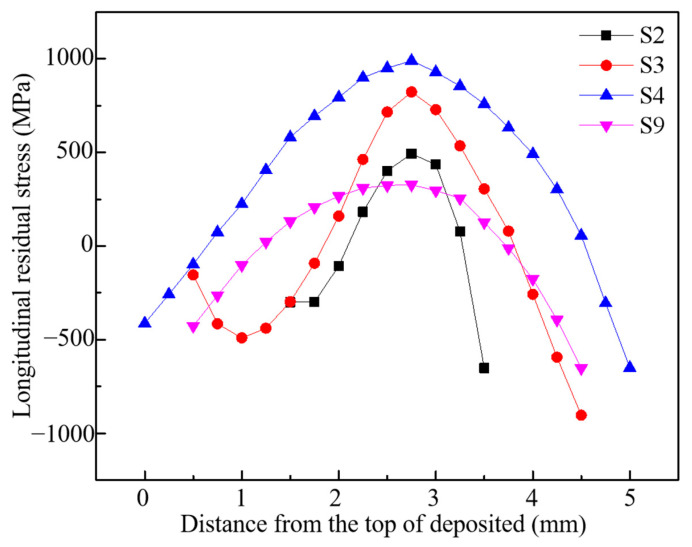
Longitudinal residual stress along the height of the deposited sample.

The distribution law of longitudinal residual stress along the height of the deposited sample is shown in Figure 11. For CW mode, the maximum longitudinal residual stress increases from 484 MPa to 990 MPa, while the laser power rises from 450 W–800 W (S2–S4). Because the increase in peak power leads to a rise in the temperature gradient of the solid region of the deposited sample, the degree of thermal expansion and cooling shrinkage of the material is more intense, resulting in an increase in thermal strain. Section 3.1 and Section 3.2 describes this in detail. It can be seen that reducing the laser power can effectively reduce and homogenize the residual stress of the deposited sample, but this method also reduces the manufacturing efficiency and increases the manufacturing cost and time. For PW mode (S9), the maximum longitudinal residual stress is about 233 MPa; it reduced by almost 70% compared to S3, which has the same energy input. Fortunately, the forming quality, density and manufacturing efficiency of the two laser modes are consistent.

## 4. Discussions

### 4.1. Temperature Gradient in Solid Region

According to temperature gradient mechanism (TGM) [31], a steep temperature gradient appeared around the molten pool due to the rapid heating of the samples’ surface and the relatively slow heat conduction. Thermal compressive strain occurs since the expansion of the top heating zone is restricted by the cold material at the bottom. When the sample cools, the metal material begins to shrink, and due to the constraint of the underlying material, this causes thermal tensile strain. When the yield strength of the material is reached, the top layer will be plastically tensile, even though it is not required for the material to become molten. A large amount of residual strain was introduced and remained in the deposited sample. The thermal strain in the solid region can be expressed by Formula (1) [6]:

(1) $$\varepsilon =-\alpha_{CTE}\left(T-T_{0}\right)=\varepsilon_{ij}^{el}+\varepsilon_{ij}^{p}+\varepsilon_{0}$$

where T represents the local temperature; $T_{0}$ represents the initial temperature; $\varepsilon_{ij}^{el}$ is the elastic strain; $\varepsilon_{ij}^{p}$ is the plastic strain; $\varepsilon_{0}$ includes the other inelastic strain, such as that from phase transformation and creep; and $\alpha_{CTE}$ is the coefficient of thermal expansion (CTE).

**Figure 12:**
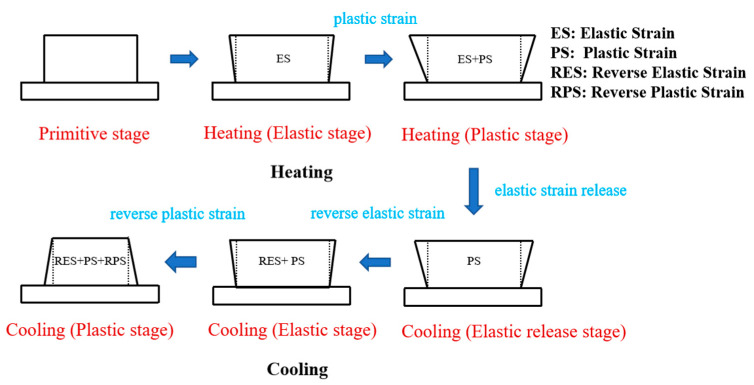
Schematic diagram of changes in thermal strain during the DED process.

The thermal strain evolution during the heating and cooling process of DED can be divided into the following five stages, as shown in Figure 12. The first stage is the elastic compression stage, where heating causes the metal material to expand outward. Due to the constraints of the surrounding cold base material, elastic compression strain is generated inside the material with a magnitude of ${\;\varepsilon }_{ij}^{th}=-\varepsilon_{ij}^{el}=-\alpha_{CTE}\left(T-T_{0}\right)$. The second stage is the plastic compression stage. After the material reaches the yield strength, it generates plastic strain. The total accumulated thermal strain of the material is the sum of elastic strain and plastic strain, and its magnitude can be expressed as $\varepsilon_{ij}^{th}=-\varepsilon_{ij}^{el}-\varepsilon_{ij}^{p}=-\alpha_{CTE}(T-T_{0}){-\varepsilon }_{ij}^{p}$. The third stage is the elastic release stage. As the laser moves, the elastic compression strain is partly released due to the cooling shrinkage effect of metal materials. The fourth stage is the elastic tension stage, in which continuous cooling causes the metal material to contract inward. Reversed tensile strain is generated internally due to the constraints of the surrounding matrix material. It reverses to generate an elastic tension strain, with the same size but opposite direction as the elastic compression stage, and can be expressed as $\varepsilon_{ij}^{th}={-\varepsilon_{ij}^{p}+\varepsilon }_{ij}^{el}={-\varepsilon }_{ij}^{p}+\alpha_{CTE}\left(T-T_{0}\right)$. The fifth stage is the plastic tension stage, where the material cools to the yield strength. This generates reversed plastic strain. At this time, the plastic tension strain generated by material cooling partly offsets the plastic compression strain generated during heating. The remaining thermal strain is the main factor contributing to the formation of residual stress inside the deposited sample. Therefore, the residual stress of the deposited sample after DED deposition is closely related to the thermal strain and temperature gradient during the deposition process.

**Figure 13:**
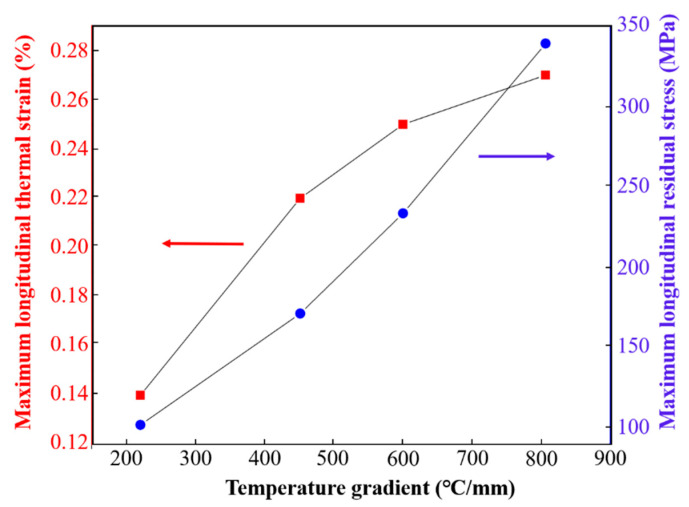
Relationship between temperature gradient, longitudinal strain (red square and arrow), residual stress (blue circle and arrow) in solid region.

Through the analysis of S1–S4 samples in Section 3, the relationship between the temperature gradient in solid region, the maximum longitudinal thermal strain and the maximum longitudinal residual stress was quantitatively established by changing the laser power, as shown in Figure 13. When the temperature gradient in the solid region increases from 320.2 °C/mm to 806.4 °C/mm, the longitudinal thermal strain increases from 0.14% to 0.26%, and the maximum longitudinal residual stress of deposited sample increases from 102.2 MPa to 339.3 MPa. The above results indicate that the mechanism of reducing residual stress by changing laser process parameters involves reasonably reducing the temperature gradient, thereby reducing the thermal strain during the deposition process.

**Figure 14:**
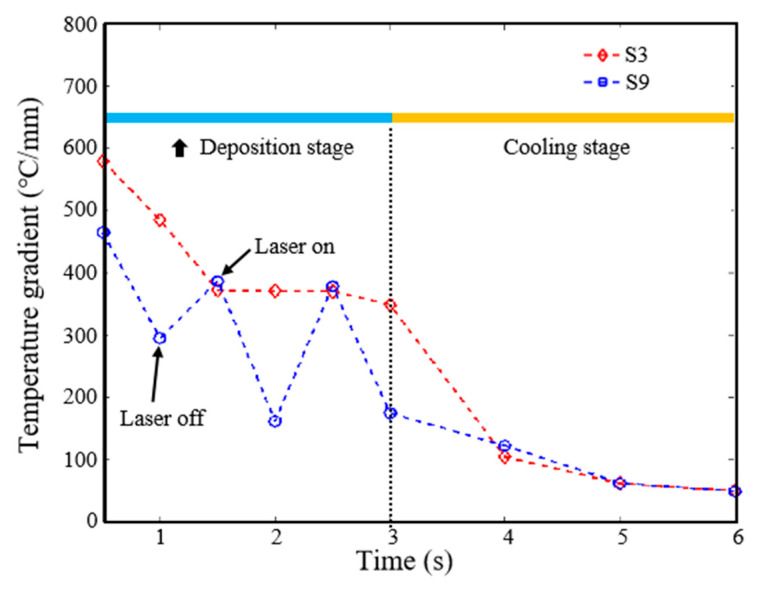
Time-dependent temperature gradient in solid region during deposition of S3 and S9 samples.

The time-dependent temperature gradient in the solid region during the deposition of S3 and S9 samples is shown in Figure 14. From the data in the figure, it can be seen that in the deposition stage, the temperature gradient in CW laser mode (S3 sample) gradually decreases from about 600 °C/mm and stabilizes at 380 °C/mm. However, the temperature gradient under PW laser mode (S9 sample) shows quasi-steady state characteristics (sawtooth). At the time of laser switching on, the temperature gradient is maintained at 380~460 °C/mm, which is slightly different from that under CW mode. When the laser is turned off, the temperature gradient drops rapidly; it is about 200~300 °C/mm. In the cooling stage, there is little difference in the temperature gradient in solid region between the two laser modes. Overall, the average temperature gradient in the solid region in PW laser mode is about 77% of that in CW laser mode (326.3 °C/mm~421.4 °C/mm). It is obvious that the temperature gradient always maintains a high level for CW mode because of the continuous laser energy input, while for PW mode, the temperature gradient presents transient characteristics caused by heat wastage during the laser-off stage [32]. A substantial decrease in temperature gradient is one of the reasons why PW mode can significantly reduce the residual stress of the deposited sample compared to CW mode under the same energy input.

### 4.2. Stress Relaxation

**Figure 15:**
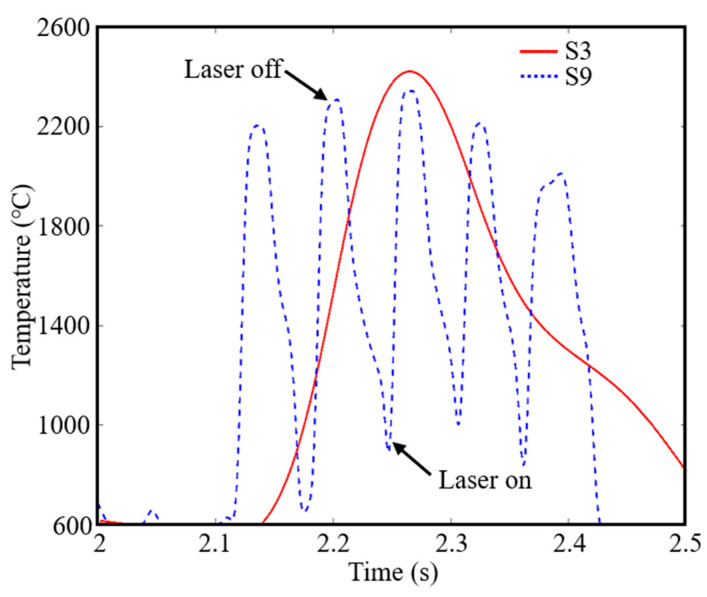
Temperature history during deposition of S3 and S9 samples.

**Figure 16:**
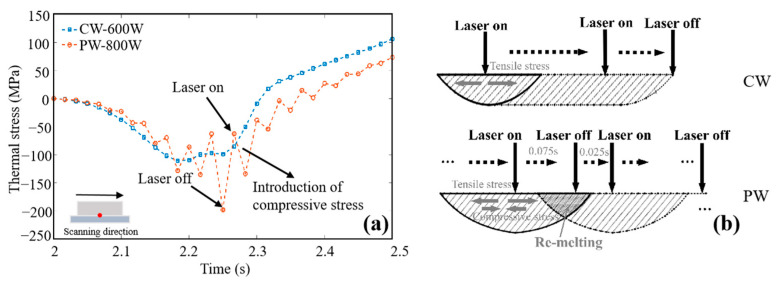
(**a**) Time-dependent thermal stress. (**b**) Schematic diagrams of thermal stress state during deposition of S3 and S9 samples.

The thermal stress generated by temperature gradient is not the unique factors that determine the final residual stress of the deposited sample [33]. Stress relaxation during the process also plays an important role in reducing the final residual stress [34]. The special thermal history in PW mode can partially release thermal stress during the deposition process, thus effectively reducing and homogenizing the residual stress of the deposited sample. The time-dependent temperature and thermal stress under CW and PW modes (S3 and S9 samples) are shown in Figure 15 and Figure 16a, and the corresponding schematic diagrams of thermal stress state under CW and PW modes (S3 and S9 samples) are shown in Figure 16b. For the CW deposited samples, when the laser approaches the measured point, the temperature of the material in the laser irradiated area rises rapidly. Due to the effect of heat conduction, a large amount of heat transfers to the area near the molten pool, resulting in the thermal expansion of the material. High temperature metal materials are limited by the underlying cold metal, which produce compressive stress. As the laser moves, the material in this area begins to cool and shrink. Similarly, tensile stress will also be generated due to the constraint of the underlying metal. Under the action of the CW laser, the material shows typical thermal expansion and cooling shrinkage. However, for PW laser, it shows jagged expansion and contraction curves in the manufacturing process, which is attributed to the cyclic temperature changes caused by periodic laser switching light. When the laser is turned on, the metal material undergoes the same expansion process as CW mode due to the increase in temperature, resulting in compressive stress. However, when the laser is turned off periodically, the temperature immediately decreases, and the material begins to shrink. Under the constraint of the underlying material, the compressive stress gradually turns into tensile stress. Fortunately, when the next laser is turned on periodically, heat is transmitted to this solidified area through heat conduction, causing the material to be reheated, which leads to the expansion trend of the metal again. Compressive stress is formed. This compressive stress partially offsets the formed tensile stress. Therefore, compared to the simple “compression-tension” thermal stress characteristics of the solid region under the CW laser mode, the thermal stress of the PW laser mode shows the serrated cyclic thermal stress characteristics of “compression-tension- compression-tension...”, which helps to reduce the residual tensile stress [35]. Moreover, there must exists a “re-melting” area in the molten pool, which would transfer heat to the cooled metal in a timely manner. It is similar to the “annealing effect”, which relieves residual stress [36].

## 5. Conclusions

In this work, a typical thermodynamic response (thermal history, thermal strain and residual stress) of 316L stainless steel in CW laser mode during DED was revealed. The effects of laser power and scanning speed on the temperature gradient, thermal strain and residual stress in solid region were studied. The quantitative relationship between temperature gradient, thermal strain and residual stress was established. On this basis, the PW laser mode is used to regulate the temperature gradient in the solid region of the deposited sample, and its periodic cyclic thermal characteristics are used to in situ release the residual stress in the material, which effectively reduces the residual stress of the deposited sample under the condition of ensuring the processing efficiency, thereby affecting quality and densification. The findings of this paper are summarized as follows:

1. The temperature gradient and thermal strain during the processing can be reduced by optimizing process parameters, especially laser power, thereby reducing the residual stress of the deposited sample effectively.
2. Although the method of changing process parameters can effectively reduce the residual stress of the deposited sample, it will bring about problems such as poor forming quality, low manufacturing efficiency and insufficient density.
3. Using the PW laser mode can significantly reduce the residual stress of the deposited sample (about 73%) because of its periodic switching light characteristics, which causes a unique cyclic temperature history, resulting in small temperature gradients and stress relaxation. The forming quality, manufacturing efficiency and density is also consistent with the traditional CW laser mode.

## Figures and Tables

**Table 1:** The main process parameters of laser additive manufacturing.

Sample No	Scanning Speed (mm/s)	Laser Power (W)	Laser Mode (mm)
S1	8	300	CW
S2	8	450	CW
S3	8	600	CW
S4	8	800	CW
S5	4	800	CW
S6	6	800	CW
S7	8	800	CW
S8	10	800	CW
S9	8	800	PW

**Table 2:** Deposition morphology and densification of S1–S4 and S9 samples.

Sample	Deposition Morphology	Mass (g)	Volume (mm^3^)	Densification
S1	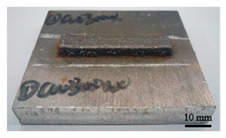	13.09	1800	92.24%
S2	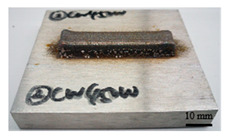	17.54	2250	99.34%
S3	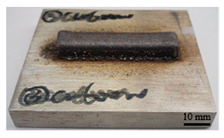	21.25	2720	99.52%
S4	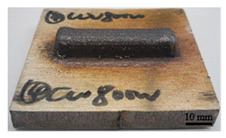	23.99	3060	99.85%
S9	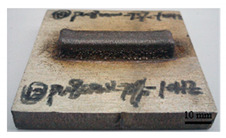	21.09	2690	99.87%

## Data Availability

Data sharing is not applicable.
